# Systematic Review and Meta-Analysis of Global Prevalence of HBsAg and HIV and HCV Antibodies among People Who Inject Drugs and Female Sex Workers

**DOI:** 10.3390/pathogens9060432

**Published:** 2020-05-31

**Authors:** Roya Rashti, Heidar Sharafi, Seyed Moayed Alavian, Yousef Moradi, Amjad Mohamadi Bolbanabad, Ghobad Moradi

**Affiliations:** 1 Social Determinants of Health Research Center, Research Institute for Health Development, Kurdistan University of Medical Sciences, Sanandaj 6617713446, Iran; Roya.rashti73@gmail.com (R.R.); amohammadi1364@gmail.com (A.M.B.); 2 Middle East Liver Diseases Center, Tehran 1598976513, Iran; heidarsharafi@gmail.com; 3 Professor of Gastroenterology and Hepatology, Middle East Liver Disease Center, Tehran 1598976513, Iran; alavian@thc.ir; 4 Department of Epidemiology, School of Public Health, Iran University of Medical Sciences, Tehran 1449614535, Iran; moradi.y@tak.iums.ac.ir

**Keywords:** human immunodeficiency virus (HIV), hepatitis C virus (HCV), hepatitis B virus (HBV), people who inject drugs (PWID), female sex workers (FSWs), Co-infection

## Abstract

The main objective of this study was to evaluate the prevalence of human immunodeficiency virus/acquired immunodeficiency syndrome (HIV/AIDS), hepatitis C virus (HCV) and hepatitis B virus (HBV) and their co-infections among people who inject drugs (PWID) and female sex workers (FSWs). Data sources were searched from January 2008 to October 2018 in different databases. Data were analyzed in Stata 16 software using the Metaprop command. The results showed that the prevalence of HIV, HCV and HBV among PWID was 15%, 60% and 6%, respectively. The prevalence of HIV, HCV and HBV among FSWs was 5%, 1% and 3%, respectively. The prevalence of HIV/HCV, HIV/HBV, HCV/HBV and HIV/HCV/HBV co-infections among PWID was 13%, 2%, 3% and 2%, respectively. The prevalence of HIV/HCV and HIV/HBV co-infections among FSWs was 3% and 1%, respectively. The results show that the prevalence of HCV and HIV infections in PWID and the prevalence of HIV in FSWs is higher than their prevalence in the general population. Interventions for the prevention of HIV and HCV in PWID appear to be poor, and may not be sufficient to effectively prevent HIV and HCV transmission.

## 1. Introduction

According to a World Health Organization (WHO) report in 2018, there are approximately 38 million people living with HIV/AIDS in the world, the largest number being in Africa, with 25.7 million [1]. The WHO’s goal is to identify 90% of HIV patients, treat 90% of those identified and virally suppress 90% of those treated by 2020 [2]. Hepatitis C virus (HCV) and hepatitis B virus (HBV) have affected 71 million and 257 million people worldwide, which can lead to cirrhosis and liver cancer, respectively. Removing these viruses, which requires significant economic and sanitary capital, will prevent more than 1.2 million deaths worldwide annually [3,4]. HCV has the highest prevalence in the Eastern Mediterranean region, followed by the European region, with a prevalence of 2.3% and 1.5%, respectively. It varies from 0.5% to 1% in other areas. HBV is most prevalent in the Western Pacific and African regions, with values of 6.2% and 6.1%, respectively. The lowest prevalence is reported in the US, with a value of 0.7% [5]. Viral hepatitis elimination program has been adapted by WHO in 2016 [6]. In line with this global program, several countries are working towards this elimination platform [7]. In this program, engagement of high-risk groups and marginal populations has been highlighted [8,9]. The efficient implementation of prevention and control measures incorporated in this program, requires detailed insight on the epidemiology of hepatitis viruses and other viral infectious agents circulating in these cohorts [9].

People who inject drugs (PWID) and female sex workers (FSWs) are key populations for blood-borne viral infections (BBVI), including HIV, HCV and HBV. PWID are usually infected through shared needles, syringes and other infected injection equipment, as well as other high-risk behaviors [10]. There are an estimated 15.6 million PWID aged 15–64 years worldwide. It is said that 23% of the new HCV cases and 33% of the annual HCV deaths are related to PWID [11,12]. According to the WHO report in 2018, HIV prevalence in PWID is estimated to be from less than 1.8% to 13.5%, but viral hepatitis has not been reported in many countries. HBV is 0.7% in some of these countries, including Afghanistan, Germany and Nepal, and 7.3% in Azerbaijan, and HCV is less than 7% in some countries, including Germany, Afghanistan and Madagascar, and more than 60% in Kazakhstan [13].

FSWs are also more exposed to high-risk behaviors, especially through sex. A study in a region with a high prevalence of HIV in India stated that about 77.5% of FSWs had drug injection history, and that they were at a higher risk of BBVI than those without drug injection. The prevalence of HIV in FSWs worldwide ranges from less than 1.4% to over 11%. The prevalence of viral hepatitis is not high in FSWs, but WHO reports that the prevalence of HBV in FSWs ranges from less than 0.3% in Brazil to over 3.6% in Peru, and hepatitis C prevalence ranges from less than 1.9% in Brazil to 6.2% in Kazakhstan in the same group. In fact, HIV is more prevalent among FSWs than HBV and HCV infections [14,15,16]. The prevalence of HIV is 22 times higher among PWID and 21 times higher among sex workers (SWs) than the general population. In 2018, about 54% of new HIV cases occurred among key populations and their sexual partners [17].

In patients co-infected with two or three HIV, HCV and HBV infections, HIV-induced immunodeficiency increases the likelihood of HBV and HCV persistence, and hepatotoxicity associated with anti-HIV treatment can worsen HBV-related liver diseases or HCV persistence. Evidence suggests that HIV infection increases the risk of HCV- and HBV-associated hepatocellular carcinoma. On the other hand, liver diseases associated with chronic HBV and HCV are leading to increased mortality and complications in HIV patients [18,19]. Adverse drug reactions (ADRs) related to antiretrovirals (ARVs) are higher in HIV patients co-infected with HBV or HCV than in HIV-monoinfected patients [20].

The purpose of this study was to conduct a systematic review and meta-analysis to estimate the prevalence of HIV (anti-HIV), HCV (anti-HCV), HBV (HBsAg) and their co-infections among PWID and FSWs, as separated by WHO geographical areas in the general population of these groups. The aim is to understand the current situation, in order to make better decisions regarding prevention, identification and treatment. 

The protocol of this study had been registered in the International Prospective Register of Systematic Reviews (PROSPERO) (CRD42018115115).

## 2. Results

### 2.1. Search Results

**Figure 1:**
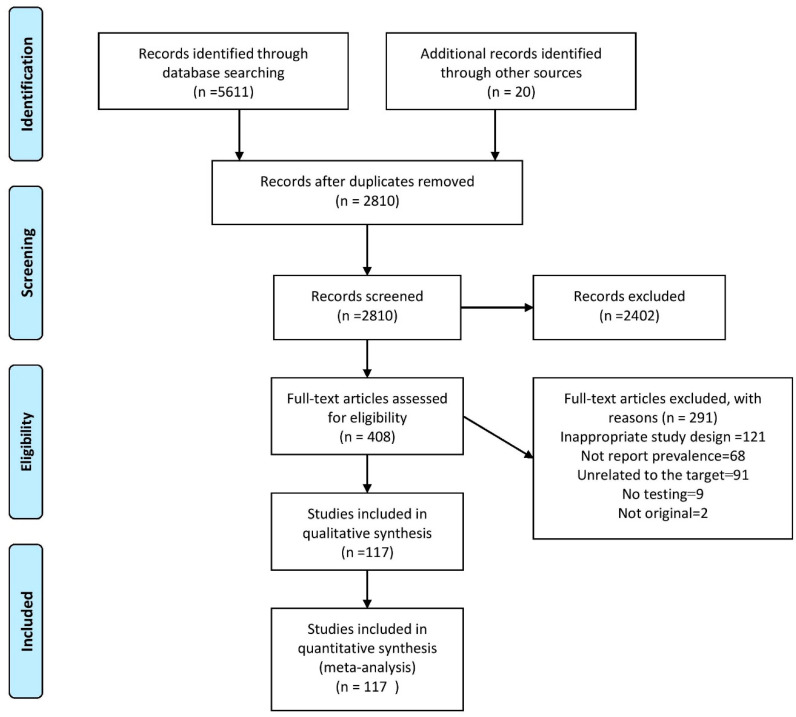
Flow-chart of systematic review of prevalence of hepatitis C virus (HCV), human immunodeficiency virus (HIV), hepatitis B virus (HBV) and their co-infections among people who inject drugs (PWID) and female sex workers (FSWs) worldwide; 2008–2018.

As showed in Figure 1, 5631 articles were found in the original search. After the removal of duplicate data, 2810 articles were selected for screening. After reviewing the title and abstract, 408 articles were selected for full text study. A total of 291 articles were excluded for various reasons, including inappropriate study design (121), failure to report prevalence (68), irrelevant target group (91), lack of proper testing (9) and non-originality (2). Eventually, after qualitative evaluation of data, 117 articles that had reported 161 records of HIV, HCV, HBV prevalence and their co-infections (HIV/HCV, HIV/HBV, HCV/HBV, HIV/HCV/HBV) among PWID and FSWs were analyzed. The characteristics of the studies are shown in Table 1. A total of 26 papers with 40 records had been published in the South-East Asia region, 25 papers with 32 records in the Americas region, 21 papers with 28 records in the Europe region, 17 papers with 19 records in the Africa region, 15 papers with 18 records in the Eastern Mediterranean region and 13 articles with 24 records in the Western Pacific region. A total of 89 papers with 119 records had been conducted on PWID and 33 papers with 42 records on FSWs.

### 2.2. Reports of Prevalence

#### 2.2.1. Prevalence of HIV among PWID and FSWs

A total of 99 articles with 133 records and a sample size of 979,659 reported HIV prevalence in PWID and FSWs, of which 74 articles with 95 records were on PWID and 29 articles with 38 records were on FSWs. The overall prevalence of HIV among PWID was estimated to be 15% (95% CI: 12–18%, *p* = 0.00, I^2^ = 99.37%) and 5% among FSWs (95% CI: 4–5%, *p* = 0.00, I^2^ = 99.41%) (Supplementary Figures S1 and S2, Table 2).

#### 2.2.2. Prevalence of HCV among PWID and FSWs

A total of 99 articles with 130 records and a sample size of 924,516 reported HCV prevalence in PWID and FSWs. A total of 76 papers with 101 records had been conducted on PWID and 24 papers with 29 records on FSWs. The overall prevalence of HCV in PWID and FSWs was 60% (95% CI: 55–64%, *p* = 0.00, I^2^ = 99.54%) and 1% (95% CI: 1–2%, *p* = 0.00, I^2^ = 97.04%), respectively (Supplementary Figures S3 and S4, Table 2).

#### 2.2.3. Prevalence of HBV among PWID and FSWs

A total of 53 articles with 64 records and a sample size of 35,007 reported HBV prevalence in PWID and FSWs. A total of 37 papers with 44 records had been conducted on PWID and 18 papers with 20 records on FSWs. The overall prevalence of HBV in PWID and FSWs was 6% (95% CI: 5–8%, *p* = 0.00, I^2^ = 94.84%) and 3% (95% CI: 1–5%, *p* = 0.00, I^2^ = 95.37%), respectively (Supplementary Figures S5 and S6, Table 2).

#### 2.2.4. Prevalence of Co-infections of HIV, HCV and HBV among PWID and FSWs

A total of 50 articles with 52 records and a sample size of 48,773 reported co-infection of HIV/HCV in PWID and FSWs. A total of 41 papers with 43 records had been conducted on PWID and nine papers with nine records on FSWs. The overall prevalence of HIV/HCV in PWID and FSWs was 13% (95% CI: 9–18%, *p* = 0.00, I^2^ = 99.36%) and 3% (95% CI: 0–9%, *p* = 0.00, I^2^ = 97.72%), respectively (Supplementary Figures S7 and S8, Table 2).

A total of 18 articles with 23 records and a sample size of 12,361 reported co-infection of HIV/HBV in PWID and FSWs, of which 12 articles with 14 records were on PWID and seven articles with nine records were on FSWs. The overall prevalence of HIV/HBV in PWID and FSWs was 2% (95% CI: 1–3%, *p* = 0.00, I^2^ = 93.26%) and 1% (95% CI: 0–3%, *p* = 0.00, I^2^ = 93.74%), respectively (Supplementary Figures S9 and S10, Table 2).

A total of 14 articles with 14 records and a sample size of 10,844 reported co-infection of HCV/HBV in PWID and FSWs, of which 13 articles with 13 records were on PWID and one article with one record was on FSWs [31]. The overall prevalence of HCV/HBV in PWID was 3% (95% CI: 1–5%, *p* = 0.00, I^2^ = 92.39%) (Supplementary Figure S11, Table 2).

A total of nine articles with nine records and a sample size of 3849 reported co-infection of HIV/HCV/HBV in PWID, but no articles reported this co-infection in FSWs. The overall prevalence of co-infection of HIV/HCV/HBV among PWID was estimated to be 2% (95% CI: 1–3%, *p* = 0.00, I^2^ = 75.19%) (Supplementary Figure S12, Table 2).

### 2.3. Subgroup Analysis by Regions of WHO

Subgroup analysis based on WHO regions is shown in Table 2. The highest prevalence of HIV in PWID is in the Africa region (24%) and the lowest prevalence is in the Eastern Mediterranean region (8%). The highest prevalence of HIV in FSWs is in the Africa (19%) and South-East Asia (18%) regions and the lowest prevalence is in Eastern Mediterranean (0%) and Western Pacific (1%) regions. The highest prevalence of HCV in PWID is in the Western Pacific (75%) and the lowest prevalence is in Africa (38%). The highest prevalence of HCV in FSWs is in Africa (9%) and the lowest prevalence is in Western Pacific and the Americas regions (1% each). The highest prevalence of HBV in PWID is in South-East Asia (9%) and the lowest prevalence is in the Americas and Eastern Mediterranean regions (1% each). The highest prevalence of HBV in FSWs is in Africa (5%) and South-East Asia (4%) regions and the lowest prevalence is in the Americas and Eastern Mediterranean (1% each). 

The highest prevalence of HIV/HCV in PWID is in the South-East Asia, Africa and Europe regions (17%, 16% and 16%, respectively) and the lowest prevalence is in the Eastern Mediterranean region (8%); the highest prevalence in FSWs is in Africa (23%) and the lowest prevalence is in the Americas and South-East Asia regions (0% and 1%, respectively). The prevalence of HIV/HBV in PWID was estimated to be 2% in South-East Asia and 1% in Africa and the Eastern Mediterranean. The highest prevalence of HIV/HBV in FSWs was in Africa with 7% and the lowest prevalence was in South-East Asia with 1%. The prevalence of HCV/HBV in PWID in the South-East Asia and Eastern Mediterranean was 3% each, and the prevalence of HIV/HCV/HBV in PWID was 2% in South-East Asia and 1% in both Africa and the Eastern Mediterranean (Table 2). 

### 2.4. Meta-Regression

Meta-regression results on heterogeneity of studies showed that sample size had a significant effect on the prevalence of HIV, HCV, HBV, HIV/HCV and HCV/HBV among PWID and HIV, HCV and HBV among FSWs, but the mean age of study participants has no significant effect (Table 3).

### 2.5. Publication Bias

Egger’s test results were significant for HIV prevalence among PWID and FSWs (*p* = 0.00), HCV among FSWs (*p* = 0.009), HBV among PWID and FSWs (*p* = 0.00), HIV/HCV among PWID (*p* = 0.00), HIV/HCV among FSWs (*p* = 0.029), HIV/HBV among PWID and FSWs (*p* = 0.003), and HCV/HBV among PWID (*p* = 0.008), pointing to publication bias. However, it was not significant for HIV/HCV/HBV prevalence (*p* = 0.432) and HCV among PWID (*p* = 0.078) indicating no publication bias.

## 3. Discussion

This systematic review and meta-analysis examined the prevalence of HIV, HCV, HBV and their co-infections among PWID and FSWs worldwide, and the results were shown by different WHO regions. The prevalence of HIV in PWID and FSWs was 15% and 5%, respectively. That means one of seven PWID and one of 20 FSWs get infected with HIV, with the highest prevalence in PWID being in Africa (24%), South-East Asia (22%) and Europe (12%), and in FSWs being in Africa (19%) and South-East Asia (18%). The number of people living with HIV worldwide in 2017 was estimated to be 36.8 million [145]. HIV is most likely transmitted through unprotected sex and syringes and needles used for injections. A total of 69.5% of HIV infection in the general population occurs through needle sharing and 10% through unprotected sex [146,147,148]. In a meta-analysis study in 2017, the prevalence of HIV among PWID worldwide was reported to be 17.8%, and the largest population under study was from sub-Saharan Africa. A total of 95% of new HIV cases were among the key populations in the Middle East and North Africa (MENA) [13]. In a 2019 study, the prevalence of HIV in PWID was 21% in Africa; in another study, HIV incidence in PWID in the MENA region was significant, with 75% of new cases occurring in PWID and their sexual partners [149,150]. The meta-analysis by Leung et al., in 2019, also estimated the highest prevalence of HIV in Africa and Asia, which is consistent with the results of our study [151]. The prevalence of HIV in Brazilian FSWs was 5%, and in part-time sex workers in Burkina Faso it was 6.5% [31,152].

The prevalence of HCV in PWID and FSWs was 60% and 1%, respectively. That means that almost one in every two PWID has HCV, with the highest prevalence of HCV in PWID being in the Western Pacific, the Americas and the Eastern Mediterranean, with 75%, 64% and 60%, respectively, and the lowest being in Africa (38%); the highest prevalence in FSWs is in Africa (9%), and the lowest prevalence is in the Americas and Western Pacific (1% each). In a 2007 meta-analysis study, the prevalence of HCV in PWID worldwide was estimated to be 50% [153]. In another study published by Nelson et al. in 2011, the prevalence of HCV in PWID was reported to be 60–80% in 25 countries and over 80% in 12 countries, with approximately 10 million PWID suffering from HCV, China and the US having the largest population [154]. Another meta-analysis study estimated the lowest prevalence of HCV was in the Middle East, North Africa, East and South-East Asia [152]. Worldwide, it is estimated that 14 million PWID are at risk for HCV exposure from contaminated blood. In 2011, the prevalence of hepatitis C in PWID worldwide was estimated to be 67%, the highest prevalence being in Eastern Europe (2.3 million) and East and South-East Asia (2.6 million) [154]. Degenhardt et al. (2017) reported a 52.3% prevalence of HCV among PWID worldwide [11]. In another European Union study, the prevalence of HCV among general population was estimated to be 0.54% to 1.50% in 2019, with the highest prevalence in PWID being in the range of 7.9–82% [155]. In a study in Brazil, the prevalence of hepatitis C in FSWs was estimated to be 0.9% [31]. The relatively high prevalence of needle/syringe sharing, low condom use, high levels of sex with sex workers, homosexual sex between men, and selling sex, indicate high-risk behaviors associated with HIV and HCV prevalence in different regions [156]. Another parameter influencing the prevalence and natural history of HCV infection is the host immune response-related genetics such as *IFNL3/4* polymorphisms which impact the spontaneous clearance of HCV, and it was also observed that the genotypes associated with favorable outcome has different prevalence in ethnic groups [157,158].

In this study, the prevalence of HBV in PWID and FSWs was 6% and 3%, respectively, with the highest prevalence in PWID being in South-East Asia (9%) and Africa and the Eastern Mediterranean (5% each), and the lowest prevalence being in the Americas and Western Pacific (1% each). Moreover, the highest prevalence in FSWs was in Africa (5%) and South-East Asia (4%), and the lowest prevalence was in the Americas and Eastern Mediterranean (1% each). Nearly 3.6% of the world’s population (257 million people) have chronic hepatitis B, with a prevalence of 0.01–2% in the UK, the US, Canada, Western Europe, and Japan, and over 8% in most sub-Saharan areas in Africa and some countries in the Western Pacific region [5,159]. In high- and middle-income countries, HBV transmission is more perinatal and horizontal. In low-income countries, however, transmission occurs through drug injection and high-risk sexual behaviors [160]. Asia and Africa have the highest HBV endemicity, but highly effective vaccination programs in some countries have pushed the pattern towards moderate or low endemicity. Therefore, China is currently the only country in Asia where HBV is of paramount importance. Countries with moderate endemicity include India, Korea, the Philippines, Taiwan and Thailand. Countries with low endemicity include Japan, Pakistan, Bangladesh, Singapore, Sri Lanka and Malaysia. Most countries in Africa have high endemicity, with the exception of Tunisia and Morocco, which have moderate endemicity [161]. HBV vaccination is effective in reducing and eliminating HBV by 2030. According to the WHO, in 2017, 97% of blood donors were screened for HBV, but there are gaps in the program and strategies have been suggested to resolve the problems, including reducing insecure injections (it has reduced from 39% in 2000 to 5% in 2010), and safer sex practices, such as minimizing the number of sexual partners and using protection (condoms). On the other hand, according to WHO, 80% of people with hepatitis live without prevention, testing and treatment [5]. In a study from Germany, the coverage of three HBV vaccines was 58% for drug users, one of the influencing factors being injection drug use [162]. The prevalence of HBsAg in PWID in the study by Nelson et al is estimated to be 5–10% in 21 countries and over 10% in 10 other countries with a population of 1.2 million [153]. In a 2017 study, the prevalence of HBV among PWID worldwide was 9.1%. In another study, its prevalence was reported to be 5% in Africa [149,154].

The prevalence of HIV/HCV in PWID and FSWs was 13% and 3%, respectively, with the highest prevalence in PWID being in South-East Asia (17%), Africa and Europe (16% each), and the lowest in Eastern Mediterranean (8%); and the highest prevalence in FSWs being in Africa, and the lowest being in the Americas and South-East Asia (23%, 0% and 0%, respectively). The prevalence of HIV/HBV in PWID and FSWs was 2% and 1%, respectively, and the prevalence of HCV/HBV and HIV/HCV/HBV in PWID was 3% and 2%, respectively. The study by Larney et al. found a positive association between the high prevalence of anti-HCV and the prevalence of HIV in PWID worldwide [163]. Co-infection with viral hepatitis in HIV-positive patients may worsen the prognosis [164]. HBV reactivation is observed in patients with HBV/HCV co-infection during HCV treatment (direct acting antivirals (DAA)) or afterwards [165].

### Strength and Limitations

Previous systematic review studies have investigated the prevalence of HIV, HCV and HBV in PWID worldwide, but we have investigated the prevalence of these infections and their co-infections in the high-risk groups PWID and FSWs as distinguished by geographical areas. One limitation of this study is the changing sensitivity of HIV, HCV and HBV diagnostic tests over time, therefore, the results of the 2018 surveys may be different from 2008. In this study, all the reviewed articles had used anti-HCV serology test to detect HCV, which does not differ between past and present infections. The articles reporting HCV-RNA were scarce and excluded. Another limitation of our study is the high heterogeneity in studies. As the analysis was performed in different geographical areas, the heterogeneity may be due to different inclusion and exclusion criteria (e.g., type of drug, minimum duration of injectable drug use, sampling location (prison, the Behavioral Disease Counseling Center, MMT Center, homeless people), frequency of sex during a specific time, number of sexual partners and condom use).

## 4. Materials and Methods 

### 4.1. Search Strategy 

This systematic review and meta-analysis was designed based on the Preferred Reporting Items for Systematic Reviews and Meta-Analyzes (PRISMA). Information sources from January 2008 to October 2018 were searched. Databases including PubMed, Scopus, Web of Science, Embase, Ovid, WHO and Google Scholar were searched, to find out the prevalence of HIV, HCV, HBV and their co-infections. The databases were searched using MESH keywords and the Boolean logic (AND, OR and NOT). The keywords included “Human Immunodeficiency Virus”, “HIV”, “Hepatitis C”, “HCV” “Hepatitis B”, “HBV”, “PWID”, “IDU”, “IVDU”, “FSWs” and “Co-infection”. 

### 4.2. Study Selection and Data Extraction 

Cross-sectional studies published in English that assessed anti-HIV, anti-HCV and HBsAg were selected. The studies that did not have a specific serology test to detect infections, those in which the prevalence rate was based on self-reports, the ones that used HBsAb, HBcAb, HBeAg and HBeAb tests to detect HBV and used HCV-RNA to detect HCV, and non-cross-sectional studies (case reports, reviews, case-control and cohort studies) were excluded. 

After consulting with experts, two authors (RR and YM) extracted the data from the original articles. Extracted data include: (1) name of first author,(2) date of publication,(3) date of study,(4) country,(5) study subjects,(6) age of patients,(7) sample size,(8) method of sampling,(9) prevalence of HIV among PWID and FSWs,(10) prevalence of HCV among PWID and FSWs,(11) prevalence of HBV among PWID and FSWs,(12) prevalence of HIV/HCV among PWID and FSWs,(13) prevalence of HIV/HBV among PWID and FSWs,(14) prevalence of HCV/HBV among PWID and FSWs,(15) prevalence of HIV/HCV/HBV among PWID and FSWs.

All the steps ranging from searching to extracting data were independently performed by two researchers. In case of disagreement between the two, the problem was solved by referring to the article, discussing the problem and, if necessary, seeking help from a third reviewer (Kappa statistic for agreement for quality assessment; 0.75).

### 4.3. Quality Assessment 

In this study, the Newcastle-Ottawa Quality Assessment Scale (NOS) checklist for cross-sectional studies was used to evaluate possible bias and quality of studies. This checklist was completed by two people. The quality of studies were judged based on such aspects as selection, comparability and outcome using the “star” rating system. Scores ranged from 0 stars (worst case) to 9 stars (best case). Studies with a score of 0–4 were categorized as low quality, 5–7 as moderate and more than 7 as high quality.

### 4.4. Statistical Analysis

Meta-analysis was performed on the eligible data to determine the prevalence of HIV/HCV, HIV/HBV, HCV/HBV, HIV/HCV/HBV, HIV, HCV and HBV in PWID and FSWs. A chi-square test was used to investigate the heterogeneity of the studies. The heterogeneity results from random-effects model were used to analyze the data at 95% confidence level. The MetaProp command was used to estimate the prevalence.

Egger’s statistical test and funnel plot were used to evaluate publication bias. Subgroup analysis was performed to investigate any qualitative confounding factors that may influence the prevalence of the disease. Subgroup analysis was conducted for the two high risk groups of PWID and FSWs in WHO geographical areas. Meta-regression was performed for mean age and sample size. All two-way statistical tests were considered with α = 0.05. Meta-analysis was performed in STATA 16.

## 5. Conclusions

The results show that the prevalence of HCV and HIV infections in PWID, and the prevalence of HIV in FSWs are higher than in the general population. The results indicate that the coverage of interventions for HIV and HCV prevention in PWID appear to be poor, and may not be sufficient to effectively prevent HIV and HCV transmission. Additionally, the lack of political commitment and, as a result, inadequate investment, reluctance to address sensitive issues related to young people’s sexual and reproductive needs and rights, and issues related to key populations and harm reduction, and a lack of systematic prevention implementation, even with regard to policy, are three interconnected reasons that seem to underpin the failure to implement effective programs at scale. Increasing the interventions for PWID and FSWs, such as HBV vaccination for the prevention of HBV, and the use of harm reduction programs, such as reducing the number of sexual partners per person, condom distribution, the use of clean needles and syringes, opiate substitution therapy (e.g., methadone) and the treatment of people living with HIV to reduce viral load and prevent onward transmission of HCV, are still a top priority in stopping the HIV and HCV epidemics. For HCV patients, education and counselling on options for care and treatment; immunization with the hepatitis A and B vaccines to prevent co-infection from these hepatitis viruses, and to protect their liver; early and appropriate medical management, including antiviral therapy; and regular monitoring for early diagnosis of chronic liver disease are necessary. Key population should be regularly monitored and screened for these infections and their associated infections.

## Figures and Tables

**Table 1:** The characteristics of studies which entered into meta-analysis.

Authors (Reference)	Year of Publication	Year of Study	Sampling Location	Study Population	Age (Mean or Median)	Method of Sampling	n/N (Prevalence of HCV)	n/N (Prevalence of HBV)	n/N (Prevalence of HIV)	n/N (Prevalence of HCV/HBV)	n/N (Prevalence of HCV/HIV)	n/N (Prevalence of HIV/HBV)	n/N (Prevalence of HCV/HIV/HBV)
**Toro-Tobón, D** [21]	2018	2014	Colombia	PWID	26	RDS	251/918 (27.3)		47/918 (5.1)				
**Rahman, M** [22]	2018	2011	Bangladesh	PWID	31.5		34/90 (37.8)						
**Rahman, M** [22]	2018	2011	Bangladesh	FSWs	31.5		0/15 (0)						
**Puga, M. A. M** [23]	2018	2009–2010	Brazil	FSWs	25	RDS	2/402 (0.5)						
**Peach, E** [24]	2018	2014	Australia	PWID	37		118/127 (92.9)	4/127 (3.1)	5/128 (3.9)				
**Patel, E. U** [25]	2018	2015–2016	India	PWID	41		213/541 (39.4)	35/541 (6.5)	86/541 (15.9)		61/541 (11.3)		
**Oyaro, M** [26]	2018	2011–2012	Kenya	PWID		RDS			19/120 (15.8)		13/120 (10.8)	1/120 (0.8)	1/120 (0.8)
**Jõgeda, E. L** [27]	2018	2011	Estonia	PWID	30	RDS	306/345 (88.7)		172/345 (49.9)		169/345 (49)		
**Jarlais, D. C. D** [28]	2018	2011–2015	USA	PWID	40		493/796 (61.9)		57/791 (7.2)				
**Iakunchykova, O** [29]	2018	2014–2015	Ukraine	PWID	36	RDS	1002/1613 (62.1)		668/1613 (41.4)		441/1613 (27.3)		
**Haussig, J. M** [30]	2018	2011–2014	German	PWID		RDS	1361/2077 (65.5)	22/2077 (1.1)	100/2077 (4.8)	20/2077 (1)		3/2077 (0.1)	
**Ferreira, O. D** [31]	2018	2016	Brazil	FSWs		RDS	38/4154 (0.9)	16/4154 (0.4)	225/4154 (5.4)	0/4154 (0)	8/4154 (0.2)	2/4154 (<0.1)	
**Duong, H. T** [32]	2018	2014	Vietnam	PWID	38	RDS	410/603 (68)		151/603 (25)				
**Demissie, M** [33]	2018	2015	Ethiopia	PWID	26	RDS	8/237 (3.4)	12/237 (5.1)	15/237 (6.3)				
**Shengelia, N** [34]	2017	2014–2015	Republic of Georgia	PWID	39	RDS			44/2022 (2.2)				
**Sharhani, A** [35]	2017	2017	Iran	PWID	36.7	snowball	332/606 (54.8)						
**Salek, T. P** [36]	2017	2012	USA	PWID (heroin)	44	randomly	64/81 (79)						
**Salek, T. P** [36]	2017	2012	USA	PWID (other narcotics)	44	randomly	41/58 (70.7)						
**Niama, F. R** [37]	2017	2011–2012	Republic of Congo	FSWs	28.31	RDS	6/805 (0.7)	34/805 (4.2)	60/805 (7.5)				
**Neaigus, A** [38]	2017	2005	USA	PWID	43	RDS	341/500 (68.2)		90/500 (18)				
**Neaigus, A** [38]	2017	2009	USA	PWID	41	RDS	390/514 (75.9)		64/514 (12.5)				
**Neaigus, A** [38]	2017	2012	USA	PWID	45	RDS	352/525 (67)		64/525 (12.2)				
**Mutagoma, M** [39]	2017	2015	Rwanda	FSWs	26	Venue-Day-Time	28/1978 (1.4)	49/1978 (2.5)	849/1978 (42.9)				
**Mmbaga, E. J** [40]	2017	2015	Tanzania	PWID	32	RDS		7/610 (1.1)	94/610 (15.4)				
**McFall, A. M** [41]	2017	2014	India	PWID (women)	30	RDS	177/796 (22.2)		421/796 (52.9)		87/796 (10.9)		
**McFall, A. M** [41]	2017	2014	India	PWID (men)	30	RDS	1721/5661 (30.4)		985/5661 (17.4)				
**Longo, J. D** [42]	2017	2013–2014	Central African Republic	FSWs	23			76/345 (22)	66/345 (19.1)			33/345 (9.6)	
**Lambdin, B. H** [43]	2017	2011–2013	Tanzania	PWID	32		359/630 (57)		187/630 (29.7)		151/630 (24)		
**Khatib, A** [44]	2017	2012	Tanzania	PWID	32	RDS	128/408 (31.4)	25/408 (6.1)	67/593 (16.4)	13/408 (3.9)	47/408 (11.5)	7/408 (1.7)	6/408 (1.5)
**Kaberg, M** [45]	2017	2013–2014	Sweden	PWID	39		1139/1386 (82.2)	29/1386 (2.1)	93/1386 (6.7)				
**Jõgeda, E. L** [46]	2017	2011	Estonia	PWID	30	RDS	306/345 (88.7)	18/345 (5.2)	172/345 (49.9)				
**Ishizaki, A** [47]	2017	2007	Vietnam	PWID	34.1			81/760 (10.7)	273/760 (35.9)			8/760 (1.1)	
**Ishizaki, A** [47]	2017	2008	Vietnam	PWID	32.5			34/302 (11.3)	81/302 (26.8)			4/302 (1.3)	
**Ishizaki, A** [47]	2017	2012	Vietnam	PWID	32.2			43/389 (11.1)	72/389 (18.5)			4/389 (1)	
**Ishizaki, A** **(47)**	2017	2007	Vietnam	FSWs	24.8			10/91 (11)	21/91 (23.1)			1/91 (1.1)	
**Ishizaki, A** [47]	2017	2008	Vietnam	FSWs	30.1			2/63 (3.2)	13/63 (20.6)			0/63 (0)	
**Ishizaki, A** [47]	2017	2012	Vietnam	FSWs	29.8			2/51 (3.9)	5/51 (9.8)			0/51 (0)	
**Handanagic, S** [48]	2017	2014–2015	Republic of Croatia (Rijeka)	PWID	34	RDS	80/255 (31.4)						
**Handanagic, S** [48]	2017	2014–2015	Republic of Croatia (Split)	PWID	37	RDS	153/399 (38.3)						
**Gupta, D** [49]	2017	2013–2014	India	PWID			80/194 (41.2)		125/194 (64.4)		51/194 (26.3)		
**de Matos, M. A** [50]	2017	2009–2010	Brazil	FSWs		RDS	3/402 (0.7)	6/402 (1.5)					
**Iversen, J** [51]	2017		Australia	PWID	40		1173/2054 (57.1)						
**Pares-Badell, O** [52]	2017	2008–2012	Spain	PWID			1578/2243 (70.4)		732/2243 (32.6)		567/2243 (25.3)		
**Wenz, B** [53]	2016	2011–2014	Germany	PWID		RDS	1361/2077 (65.5)		101/2077 (4.9)		84/2077 (4)		
**Solomon, S. S** [54]	2016	2005	India	PWID	39		355/998 (35.6)	71/998 (7.1)	148/998 (14.8)	26/998 (2.6)	103/998 (10.3)		
**Skocibusic, S** [55]	2016		Bosnia and Herzegovina	PWID		randomly	63/120 (52.5)	1/120 (0.8)					
**Nielsen, S** [56]	2016	2011–2014	Germany	PWID		RDS	854/2077 (41.1)		100/2077 (4.8)				
**Kermode, M** [57]	2016	2009–2010	India	PWID	29.8	RDS	607/821 (73.9)		253/821 (30.8)		241/821 (29.4)		
**Hsieh, M. H** [58]	2016	2008–2010	Taiwan	PWID	36.2		517/566 (91.3)	87/566 (15.4)	301/566 (53.2)				
**Handanagic, S** [59]	2016	2014–2015	Croatia (Zagreb)	PWID		RDS	55/176 (31.3)		1/176 (0.6)				
**Handanagic, S** [59]	2016	2014–2015	Croatia (Rejika)	PWID		RDS	85/254 (33.5)		2/254 (0.8)				
**Handanagic, S** [59]	2016	2014–2015	Croatia (Split)	PWID		RDS	173/387 (44.7)		1/390 (0.3)				
**Fotiou, A** [60]	2016	2013	Greece	PWID	36		447/563 (79.4)		88/562 (15.7)		81/541 (15)		
**Folch, C** [61]	2016	2010–2011	Spain	PWID			548/761 (72)		253/761 (33.2)				
**Fernandez-Lopez, L** [62]	2016	2011	Spain	PWID	35.6		35/172 (20.3)		5/198 (2.5)				
**Des Jarlais, D. C** [63]	2016	2014	Vietnam	PWID	37	RDS	403/603 (66.8)		152/603 (25.2)				
**Chen, Yi M. D** [64]	2016	2010–2015	China (high tier)	FSWs	35		12/1832 (0.7)		3/1832 (0.2)				
**Chen, Yi M. D** [64]	2016	2010–2015	China (middle tier)	FSWs	35		51/9938 (0.5)		38/9938 (0.4)				
**Chen, Yi M. D** [64]	2016	2010–2015	China (low tier)	FSWs	35		79/10,686 (0.7)		150/10,686 (1.4)				
**Blackburn, N. A** [65]	2016	2012–2014	USA	PWID	37		3495/15,274 (22.9)		576/15,274 (3.8)		166/15,274 (1.1)		
**Abadie, R** [66]	2016	2015	Puerto Rico	PWID	41.8	RDS					19/315 (6)		
**Bouscaillou, J** [67]	2016	2014	Ivory Coast	PWID	33.5	RDS			4/73 (5.5)				
**Bouscaillou, J** [67]	2016	2014	Ivory Coast	FSWs	33.5	RDS			9/49 (18.4)				
**Tun, W** [68]	2015	2011	Kenya	PWID	31	RDS			50/269 (18.6)				
**Tang, Z. Z** [69]	2015	2010	China	FSWs	28		128/12,622 (1)		125/12,622 (1)				
**Rosinska, M** [70]	2015	2004–2005	Poland	PWID	26 29	snow-ball	448/763 (58.7)		137/763 (18)		130/763 (17)		
**Mwatelah, R. S** [71]	2015		Kenya	PWID	33		25/152 (16.4)		159/186 (85.5)		24/152 (15.8)		
**Kurth, A. E** [72]	2015	2012	Kenya (Nairobi)	PWID	31.71	RDS			96/663 (14.5)				
**Kurth, A. E** [72]	2015	2012	Kenya (Coast region)	PWID	30.40	RDS			230/1122 (20.5)				
**Jordan, A. E** [73]	2015	2006–2013	USA	PWID	41.2		1047/1535 (68.2)		183/1535 (11.9)		159/1535 (10.4)		
**Iversen, J** [74]	2015	2004–2013	Australia	PWID	34		2879/5378 (53.5)		29/5378 (0.5)				
**Fan, Y. G** [75]	2015	2012–2013	China	FSWs			44/622 (7.1)		7/622 (1.1)				
**Collier, M. G** [76]	2015	2009–2010	USA	PWID	26		135/519 (26)	7/519 (1.3)					
**Bugssa, G** [77]	2015	2013	Ethiopia	FSWs	32	randomly		19/319 (6)	38/319 (11.9)				
**Al-Tayyib, A. A** [78]	2015	2009	USA (Colorado state)	PWID		RDS	289/395 (73.2)		18/395 (4.6)		15/395 (3.8)		
**Al-Tayyib, A. A** [78]	2015	2009	USA (Washington state)	PWID		RDS	189/260 (72.7)		15/260 (5.8)		11/260 (4.2)		
**Zibbell, J. E** [79]	2014	2012	USA	PWID	30		34/100 (34)						
**Zhang, L** [80]	2014	2004	China	FSWs	24				2/343 (0.6)				
**Zhang, L** [80]	2014	2010	China	FSWs	26				6/404 (1.5)				
**Wang, L** [81]	2014	2008	China	FSWs		randomly			404/67,296 (0.6)				
**Wang, L** [81]	2014	2009	China	FSW		randomly	1328/147,528 (0.9)		590/147,528 (0.4)				
**Wang, L** [81]	2014	2010	China	FSWs		randomly	1596/199,571 (0.8)		599/199,571 (0.3)				
**Wang, L** [81]	2014	2011	China	FSWs		randomly	1434/204,873 (0.7)		615/204,873 (0.3)				
**Wang, L** [81]	2014	2012	China	FSWs		randomly	1662/207,811 (0.8)		623/207,811 (0.3)				
**Ruisenor-Escudero, H** [82]	2014	2009	Afghanistan	PWID	28	RDS	221/548 (40.3)	39/548 (7.1)	39/548 (7.1)		37/548 (6.8)		
**Ramezani, A** [83]	2014	2012	Iran	PWID	33.3		56/100 (56)	6/100 (6)	19/100 (19)		15/100 (15)		
**Palmateer, N. E** [84]	2014	2008–2009	Scotland	PWID	33.6		1420/2629 (54)						
**Palmateer, N. E** [84]	2014	2010	Scotland	PWID	34.6		1774/3168 (56)						
**Palmateer, N. E** [84]	2014	2011–2012	Scotland	PWID	35.3		1142/2154 (53)						
**Li, L** [85]	2014	2012	China	PWID	36.2	RDS	154/370 (41.6)		68/370 (18.4)				
**Javadi, A** [86]	2014	2008–2009	Iran	PWID	35.3	simple			6/539 (1.1)		6/539 (1.1)		
**Hsieh, M. H** [87]	2014	2008–2010	Taiwan	PWID	36.1		513/562 (91.3)	86/562 (15.3)	297/562 (52.8)	78/562 (13.9)	293/562 (52.1)	56/562 (10)	
**Broz, D** [88]	2014	2009	USA	PWID		RDS			397/9652 (4.1)				
**Goswami, P** [89]	2014	2008	India (Manipour)	PWID		RDS	565/839 (67.3)	51/839 (6.1)	233/839 (27.8)				
**Goswami, P** [89]	2014	2009	India (Manipour)	PWID		RDS	601/860 (69.9)	92/860 (10.7)	251/860 (29.2)				
**Goswami, P** [89]	2014	2008	India (Nagaland)	PWID		RDS	121/821 (14.7)	46/821 (5.6)	12/821 (1.5)				
**Goswami, P** [89]	2014	2009	India (Nagaland)	PWID		RDS	125/829 (15.1)	67/829 (8.1)	13/829 (1.6)				
**Seaberg, E. C** [90]	2014		USA	PWID			280/652 (42.9)	43/640 (6.7)	410/652 (62.9)	13/640 (2)	181/652 (27.8)		
**Zhou, Y. J** [91]	2013	2010	China	FSWs			128/12,622 (1)		125/12,622 (1)				
**Valadez, J. J** [92]	2013	2010	Libya	FSWs		RDS	5/69 (7.2)	2/69 (2.9)	7/69 (10.1)		3/69 (4.3)	0/69 (0)	
**Taylor, A** [93]	2013	2010–2011	Scotland	PWID	34		495/1625 (30.5)						
**Schuelter-Trevisol, F** [94]	2013	2009	Brazil	FSWs	28		13/147 (8.8)		13/147 (8.8)		3/147 (2)		
**Salter, M. L** [95]	2013	2013	USA	PWID	47		1025/1191 (86.1)				322/1191 (27)		
**Prasetyo, A. A** [96]	2013	2009	Indonesia	PWID	32.3		47/94 (50)	1/94 (1.1)	13/94 (13.8)	0/94 (0)	12/94 (12.8)	0/94 (0)	
**Praseeda, S. D** [16]	2013	2007–2010	India	FSWs	31		7/250 (2.8)	19/250 (7.6)	105/250 (42.4)		3/250 (1.2)	7/250 (2.8)	
**Javadi, A** [97]	2013	2008–2009	Iran	PWID	35.3	census			6/539 (1.1)				
**Huik, K** [98]	2013	2006–2007	Estonia	PWID	26	RDS	281/373 (75.3)		205/373 (55)		174/373 (46.6)		
**Hakre, S** [99]	2013	2009–2011	Republic of Panama	FSWs	29.4	Venue-Day-Time	2/999 (0.2)	6/999 (0.6)	7/999 (0.7)				
**Garfein, R. S** [100]	2013	2009–2010	USA	PWID	28	RDS	137/510 (26.9)		21/510 (4.1)		6/510 (1.2)		
**Chalana, H** [101]	2013	2009–2012	India	PWID	30.4		78/118 (66.1)	6/118 (5.1)	18/118 (15.2)	2/118 (1.7)	12/118 (10.2)	2/118 (1.7)	
**Bowring, A. L** [102]	2013	2011	Tanzania	PWID	30	snowball and targeted	74/267 (27.7)		93/267 (34.8)		45/267 (16.9)		
**Basu, D** [103]	2013	2008–2010	India	PWID	31.2		47/103 (45.6)	2/103 (1.9)					
**Alipour, A** [104]	2013	2010	Iran	PWID	37	convenience	86/226 (38.1)	8/226 (3.5)	21/226 (9.3)				
**Kotaki, T** [105]	2013	2012	Indonesia	FSWs	32	randomly	1/197 (0.5)	8/197 (4.1)	22/197 (11.2)		0/197 (0)	0/197 (0)	
**Min, J. A** [106]	2013	2007–2010	Korea	PWID	41.9		154/318 (48.4)	21/318 (6.6)	0/318 (0)	13/318 (4.1)			
**Johnston, L. G** [107]	2013	2012	Republic of Mauritius	FSWs	31	RDS	140/295 (47.5)	0/295 (0)	97/297 (32.7)		84/295 (28.5)		
**Yen, Y. F** [108]	2012	2006–2010	Taiwan	PWID	40		1318/1447 (91.1)		194/1447 (13.4)		190/1447 (13.1)		
**Sofian, M** [109]	2012	2009	Iran	PWID	30.7		91/153 (59.5)	11/153 (7.2)	9/153 (5.9)	9/153 (5.9)	8/153 (5.2)	3/153 (2)	2/153 (1.3)
**Goldenberg, S. M** [110]	2012	2008–2010	Mexico	FSWs	33				35/624 (5.6)				
**Ghosh, I** [111]	2012		India	FSWs				0/45 (0)	7/45 (15.6)				
**Ghosh, I** [111]	2012		India	PWID				2/58 (3.4)	2/58 (3.4)				
**Dunford, L** [112]	2012	2008–2009	Vietnam	PWID	45.7		556/1000 (55.6)						
**Dunford, L** [113]	2012	2008–2009	Vietnam	PWID				174/1000 (17.4)				49/1000 (4.9)	44/1000 (4.4)
**Barua, P** [114]	2012	2006	India	FSWs		RDS	41/426 (9.6)		57/426 (13.4)		21/426 (4.9)		
**Wu, N** [115]	2011	2009	China	PWID			115/141 (81.6)						
**Pilon, R** [116]	2011	2007	Canada	PWID		chain-referral	246/407 (60.4)		41/407 (10.1)		40/407 (9.8)		
**Mir-Nasseri, M. M** [117]	2011	2001–2002	Iran	PWID	35.24	randomly	359/518 (69.3)	19/518 (3.7)	70/452 (15.5)	16/518 (3.1)	58/452 (12.8)	3/452 (0.7)	3/452 (0.7)
**Kassak, K** [118]	2011	2007–2008	Lebanon	FSWs		RDS	0/103 (0)	0/103 (0)	0/103 (0)				
**Kassaian, N** [119]	2011	2009–2010	Iran	FSWs	30.84	snowball	9/91 (9.9)	1/91 (1.1)					
**Johnston, L** [120]	2011	2009	Mauritius	PWID	31	RDS	495/511 (96.9)	39/511 (7.6)	230/511 (45)				
**Chang, S. Y** [121]	2011	2006–2009	Taiwan	PWID	37			36/211 (17.1)					
**Znazen, A** [122]	2011	2007	Tunisia	FSWs	34		2/183 (1.1)	1/183 (0.5)	0/183 (0)				
**Telan, E. F. O** [123]	2011	2007	Philippines	PWID		RDS	219/250 (87.6)		1/250 (0.4)				
**Telan, E. F. O** [123]	2011	2009	Philippines	PWID		RDS	323/341 (94.7)		2/341 (0.6)				
**Telan, E. F. O** [123]	2011	2010	Philippines	PWID		RDS	59/59 (100)		44/59 (74.6)				
**Todd, C. S** [124]	2010	2006–2008	Afghanistan	FSWs	29		10/520 (1.9)	34/520 (6.5)	1/520 (0.2)				
**Plitt, S. S** [125]	2010	2005	Canada	PWID	38		181/274 (66.1)		65/272 (23.9)		62/272 (22.8)		
**Mahfoud, Z** [126]	2010	2007–2008	Lebanon	FSWs		RDS			0/95 (0)				
**Mahfoud, Z** [126]	2010	2007–2008	Lebanon	PWID		RDS	43/81 (53.1)	2/81 (2.5)	0/81 (0)				
**Iversen, J** [127]	2010	1998–2008	Australia	PWID	31		8100/15,583 (52)						
**Alavi, S. M** [128]	2010	2002–2006	Iran	PWID	24.8		103/333 (30.9)	12/333 (3.6)	60/333 (18)				
**Shethwala, N. D** [129]	2009		India	FSWs				10/300 (3.3)	35/300 (11.7)			2/300 (0.7)	
**Rehan, N** [130]	2009	2004	Pakistan (Karachi)	PWID		RDS	347/399 (87)						
**Rehan, N** [130]	2009	2004	Pakistan (Lahore)	PWID		RDS	348/380 (91.6)						
**Mahanta, J** [131]	2009	2004–2006	India	PWID	26		190/398 (47.7)	15/397 (3.8)	43/398 (10.8)	8/398 (2)	34/398 (8.5)		3/398 (0.8)
**Lidman, C** [132]	2009	2004–2006	Sweden	PWID	35.6		268/310 (86.5)	8/310 (2.6)	3/310 (1)				
**Dumchev, K. V** [133]	2009	2005	Ukraine	PWID	28.9	snowball	230/315 (73)		44/315 (14)		38/315(12.1)		
**Davoodian, P** [134]	2009	2002	Iran	PWID	35.4	randomly	163/249 (65.5)	12/249 (4.8)	38/249 (15.3)	7/249 (2.8)	36/249 (14.5)	3/249 (1.2)	3/249 (1.2)
**Chu, F. Y** [135]	2009	2005	Taiwan	PWID	32.4		172/192 (89.6)	32/192 (16.7)	49/192 (25.5)				
**Uuskula, A** [136]	2008	2005–2006	Estonia	FSWs	29.5	RDS	15/191 (7.9)		16/206 (7.8)		5/185 (2.7)		
**Tseng, F. C** [137]	2008	1998–2000	USA	PWID	45		2092/2296 (91.1)	73/2296 (3.2)	273/2296 (11.9)				
**Sunthornchart, S** [138]	2008	2003–2005	Thailand	PWID					551/1535 (35.9)				
**Solomon, S. S** [139]	2008	2004–2005	India	PWID	35	convenience	566/912 (62.1)	101/912 (11.1)	271/912 (29.7)		235/912 (25.8)		25/912 (2.7)
**Ngo, T. D** [140]	2008	2004	China	FSWs	26		24/310 (7.7)		12/310 (3.9)		8/310 (2.6)		
**Neaigus, A** [141]	2008	2004–2006	USA (Newark)	PWID	32.8		169/205 (82.4)		52/199 (26.1)				
**Neaigus, A** [141]	2008	2004–2006	USA (NYC)	PWID	32.8		151/282 (53.5)		15/288 (5.2)				
**Kuniholm, M. H** [142]	2008	1997–1998	Georgia	PWID			539/926 (58.2)	67/926(7.2)	5/926 (0.5)		4/926 (0.4)		
**Jindal, N** [143]	2008		India	PWID			53/157 (33.8)	28/157(17.8)	26/157 (16.6)	2/157 (1.3)	11/157 (7)	2/157 (1.3)	2/157 (1.3)
**Baumbach, J. P** [144]	2008	2005	USA (Mexico)	PWID	38.3	RDS	194/203 (95.6)		6/203 (3)				
**Baumbach, J. P** [144]	2008	2005	USA (Texas)	PWID	42	RDS	122/147 (83)		9/155 (5.8)				
**Baumbach, J. P** [144]	2008	2005	USA (New Mexico)	PWID	42	RDS	76/95 (80)		1/100 (1)				

RDS, respondent-driven sampling; PWID, people who injection drugs; FSWs, female sex workers.

**Table 2:** The prevalence of HIV, HCV, HBV and their co-infections among PWID and FSWs by regions of the World Health Organization (WHO); 2008–2018.

Prevalence	Americas, % (95% CI)	Africa, % (95% CI)	South-East Asia, % (95% CI)	Europe, % (95% CI)	Eastern Mediterranean, % (95% CI)	Western Pacific, % (95% CI)	Total, % (95% CI)
**PWID**							
**HIV**	10 (7–14)	24 (16–34)	22 (16–28)	12 (6–20)	8 (3–13)	11 (2–26)	15 (12–18)
**HCV**	64 (51–77)	38 (10–72)	54 (43–66)	59 (53–65)	60 (46–73)	75 (68–82)	60 (55–64)
**HBV**	3 (1–6)	5 (2–9)	9 (7–11)	3 (1–5)	5 (4–6)	-	6 (5–8)
**HIV/HCV**	9 (3–18)	16 (11–22)	17 (11–24)	16 (16–28)	8 (4–14)	-	13 (9–18)
**HIV/HBV**	-	1 (1–3)	2 (1–5)	-	1 (0–2)	-	2 (1–3)
**HBV/HCV**	-	-	3 (1–6)	-	3 (2–5)	-	3 (1–5)
**HIV/HCV/HBV**	-	1 (0–2)	2 (1–4)	-	1 (0–2)	-	2 (1–3)
**FSWs**							
**HIV**	4 (2–9)	19 (8–34)	18 (10–27)	-	0 (0–0)	1 (0–1)	5 (4–5)
**HCV**	1 (0–2)	9 (0–29)	3 (0–8)	-	2 (0–5)	1 (1–1)	1 (1–2)
**HBV**	1 (0–1)	5 (1–10)	4 (2–7)	-	1 (0–6)	-	3 (1–5)
**HIV/HCV**	0 (0–0)	23 (18–27)	1 (0–6)	-	-	-	3 (0–9)
**HIV/HBV**	-	7 (5–10)	1 (0–2)	-	-	-	1 (0–3)

**Table 3:** The results of met-regression on heterogeneity of pooled estimations.

Study Population		Coefficient	[95% Confidence Interval]		Standard Error	*t*	*p* > t
**PWID**	**HIV**						
	**age**	0.27	−0.12	0.67	0.19	1.39	0.17
	**Sample size**	0.19	0.15	0.23	0.02	9.47	0.00
	**HCV**						
	**age**	0.07	−0.29	0.45	0.18	0.43	0.67
	**Sample size**	0.59	0.54	0.64	0.02	23.96	0.00
	**HBV**						
	**age**	0.05	−0.12	0.24	0.08	0.69	0.5
	**Sample size**	0.06	0.04	0.09	0.01	6.26	0.00
	**HIV/HCV**						
	**age**	0.18	−0.30	0.66	0.22	0.81	0.43
	**Sample size**	0.16	0.12	0.20	0.02	7.76	0.00
	**HIV/HBV**						
	**age**	−0.15	−0.58	0.27	0.17	−0.90	0.40
	**Sample size**	0.02	0.00	0.04	0.01	2.02	0.06
	**HBV/HCV**						
	**age**	0.00	−0.51	0.50	0.18	−0.01	0.99
	**Sample size**	0.04	0.00	0.07	0.01	2.75	0.02
	**HIV/HCV/HBV**						
	**age**	0.04	−0.89	0.98	0.61	0.7	0.65
	**Sample size**	0.00	−0.01	0.01	0.00	0.03	0.97
**FSW** **s**	**HIV**						
	**age**	−0.38	−1.16	0.38	0.35	−1.10	0.29
	**Sample size**	0.10	0.06	0.14	0.02	5.27	0.00
	**HCV**						
	**age**	−0.01	−0.38	0.34	0.15	−0.11	0.91
	**Sample size**	0.05	0.01	0.08	0.01	2.71	0.01
	**HBV**						
	**age**	0.23	−0.10	0.57	0.14	1.66	0.14
	**Sample size**	0.05	0.02	0.08	0.01	3.65	0.00
	**HIV/HCV**						
	**age**	0.12	−1.55	1.80	0.13	0.96	0.51
	**Sample size**	0.06	−0.02	0.16	0.03	1.73	0.13
	**HIV/HBV**						
	**Sample size**	0.03	−0.03	0.10	0.02	1.61	0.20

## Supplementary Materials

The following are available online at https://www.mdpi.com/2076-0817/9/6/432/s1, Figure S1. Global prevalence of HIV among PWID worldwide; 2008–2018. Figure S2. Global prevalence of HIV among FSWs worldwide; 2008–2018. Figure S3. Global prevalence of HCV among PWID worldwide; 2008–2018. Figure S4. Global prevalence of HCV among FSWs worldwide; 2008–2018. Figure S5. Global prevalence of HBV among PWID worldwide; 2008–2018. Figure S6. Global prevalence of HBV among FSWs worldwide; 2008–2018. Figure S7. Global prevalence of HIV/HCV co-infection among PWID worldwide; 2008–2018. Figure S8. Global prevalence of HIV/HCV co-infection among FSWs worldwide; 2008–2018. Figure S9. Global prevalence of HIV/HBV co-infection among PWID worldwide; 2008–2018. Figure S10. Global prevalence of HIV/HBV co-infection among FSWs worldwide; 2008–2018. Figure S11. Global prevalence of HCV/HBV co-infection among PWID worldwide; 2008–2018. Figure S12. Global prevalence of HIV/HCV/HBV co-infection among PWID worldwide; 2008–2018.
