# Supplementary material for: Systematic Review and Meta-Analysis of Global Prevalence of HBsAg and HIV and HCV Antibodies among People Who Inject Drugs and Female Sex Workers
Source: Pathogens. 2020 May 31;9(6):432. doi: 10.3390/pathogens9060432 (PMC7350380; doi:10.3390/pathogens9060432)
Supplement: Supplementary file 1 [file pathogens-09-00432-s001.pdf]

Supplementary Materials: Figure S1. Global prevalence of HIV among PWID worldwide; 2008–2018. Figure S2. Global prevalence of HIV among FSWs worldwide; 2008–2018. Figure S3. Global prevalence of HCV among PWID worldwide; 2008–2018. Figure S4. Global prevalence of HCV among FSWs worldwide; 2008–2018. Figure S5. Global prevalence of HBV among PWID worldwide; 2008–2018. Figure S6. Global prevalence of HBV among FSWs worldwide; 2008–2018. Figure S7. Global prevalence of HIV/HCV co-infection among PWID worldwide; 2008–2018. Figure S8. Global prevalence of HIV/HCV co-infection among FSWs worldwide; 2008–2018. Figure S9. Global prevalence of HIV/HBV co-infection among PWID worldwide; 2008–2018. Figure S10. Global prevalence of HIV/HBV co-infection among FSWs worldwide; 2008–2018. Figure S11. Global prevalence of HCV/HBV co-infection among PWID worldwide; 2008–2018. Figure S12. Global prevalence of HIV/HCV/HBV co-infection among PWID worldwide; 2008–2018.

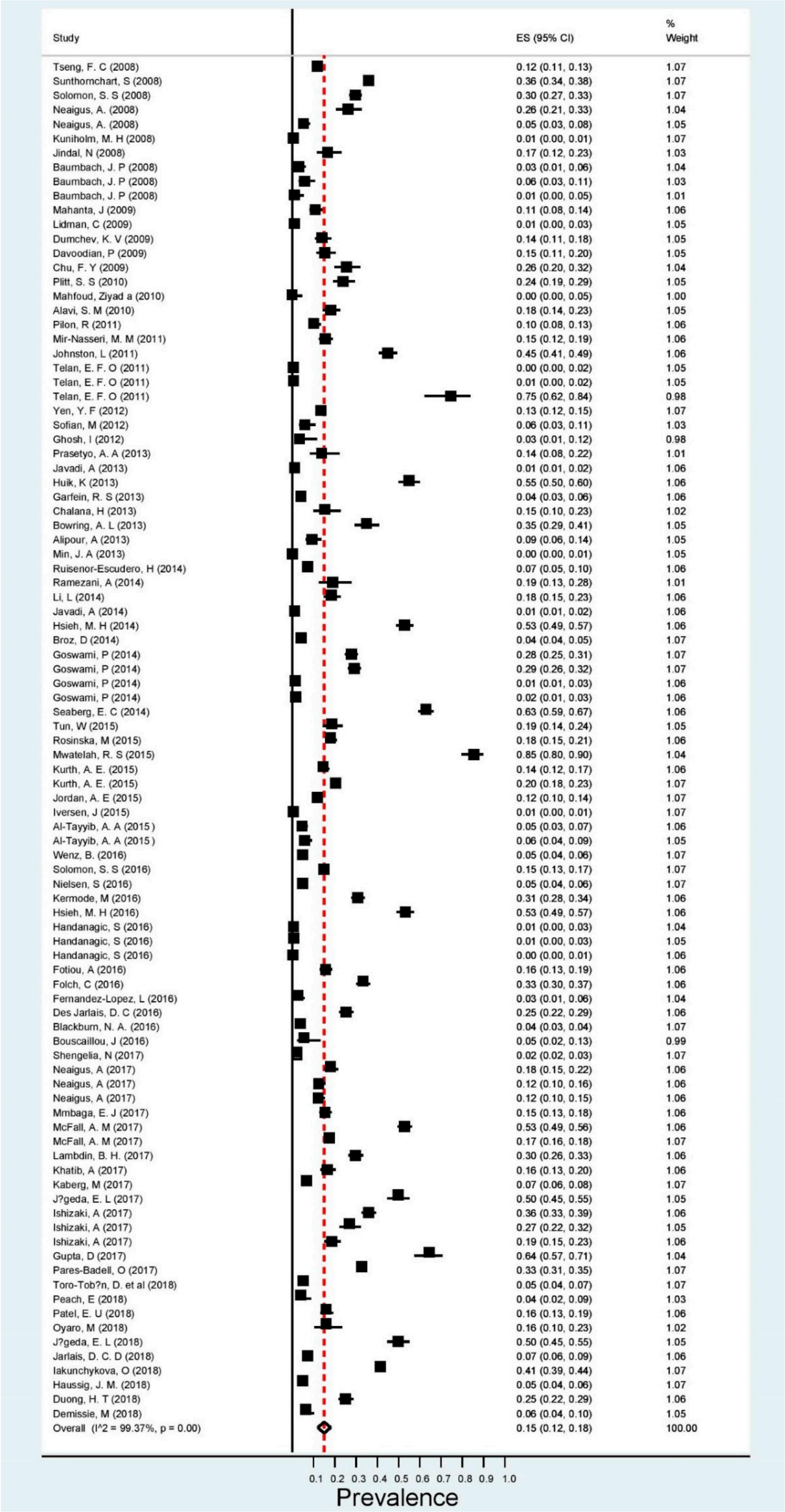

Figure S1. Global prevalence of HIV among PWID worldwide; 2008–2018.

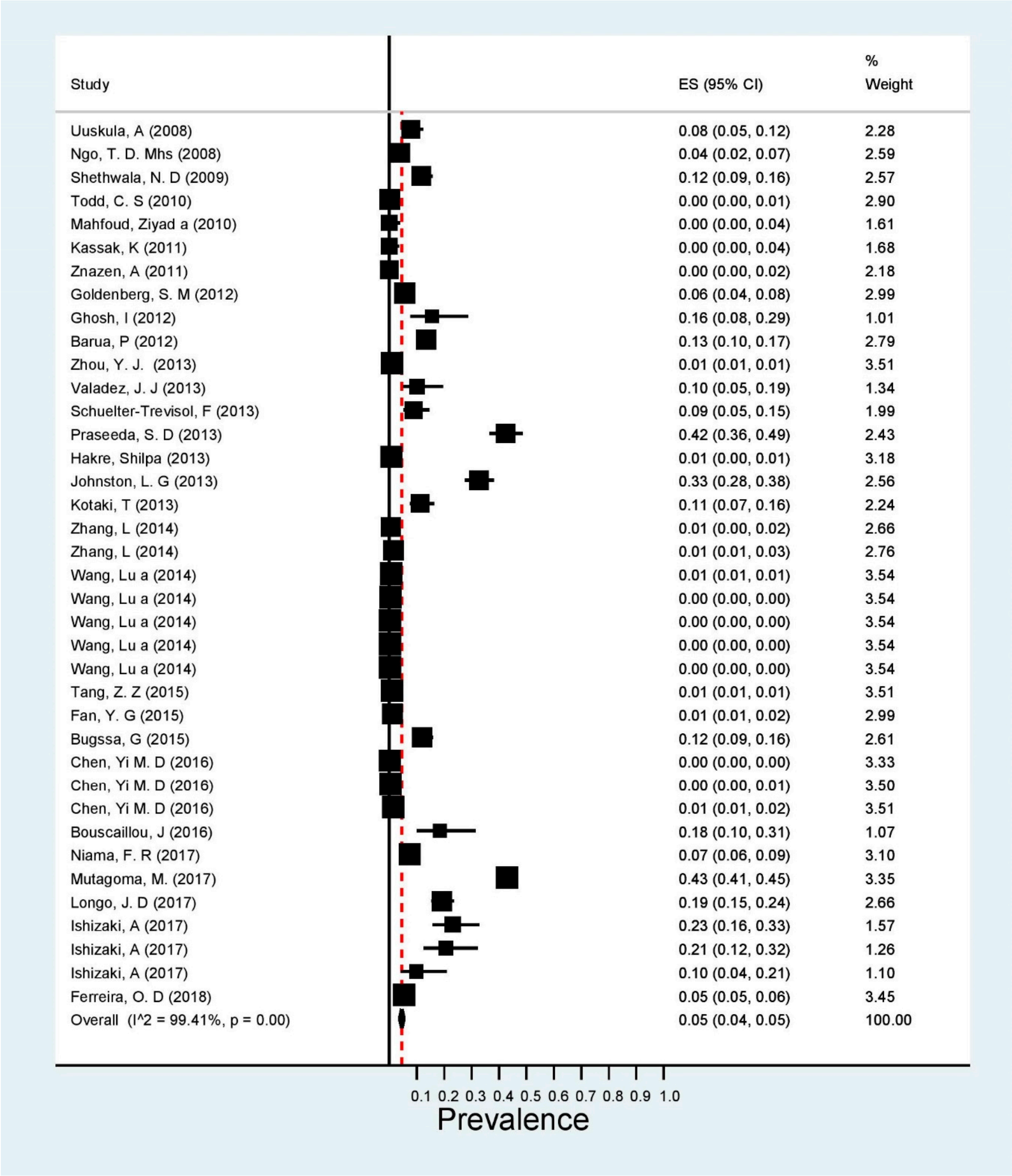

Figure S2. Global prevalence of HIV among FSWs worldwide; 2008–2018.

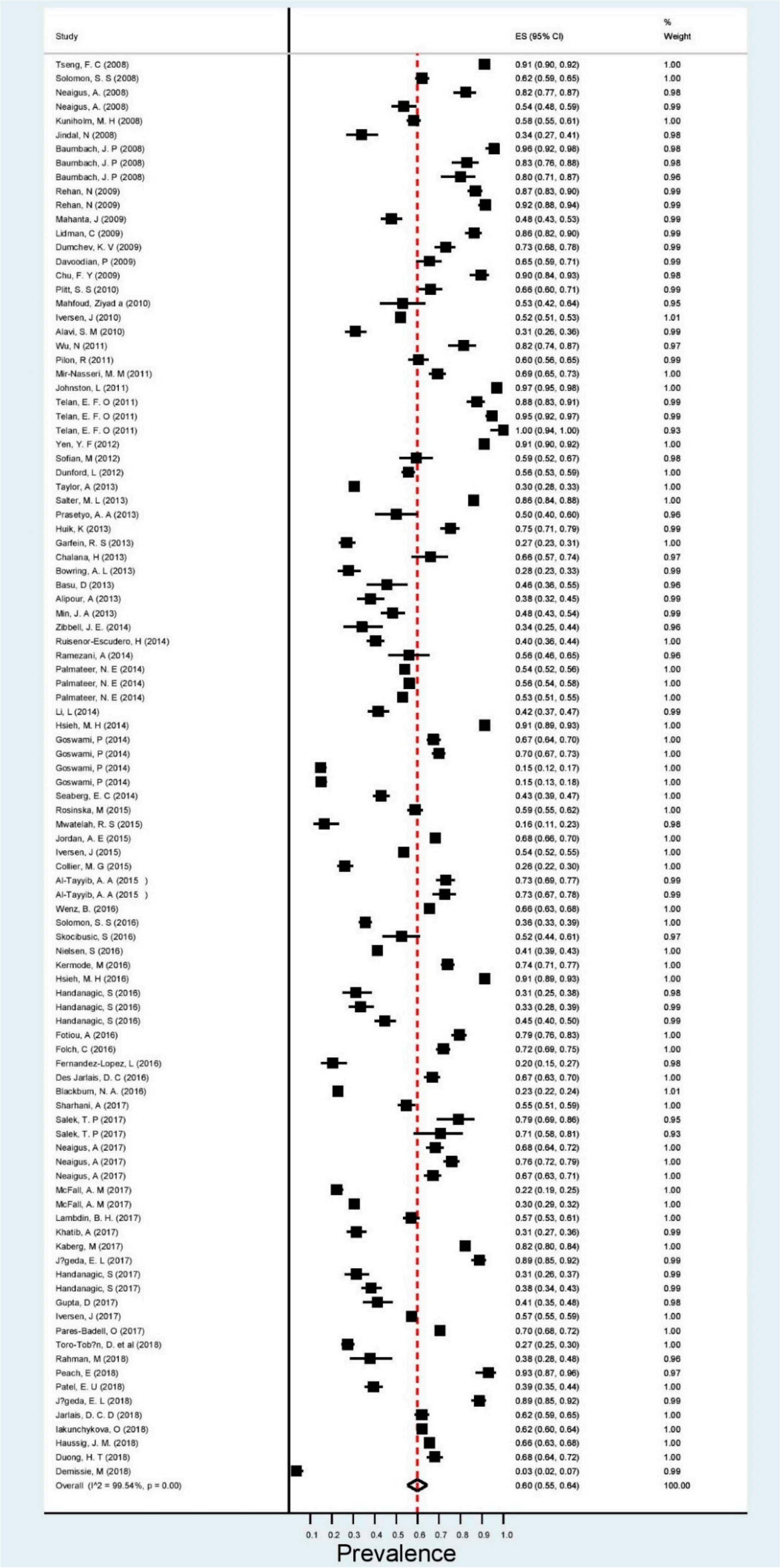

Figure S3. Global prevalence of HCV among PWID worldwide; 2008–2018.

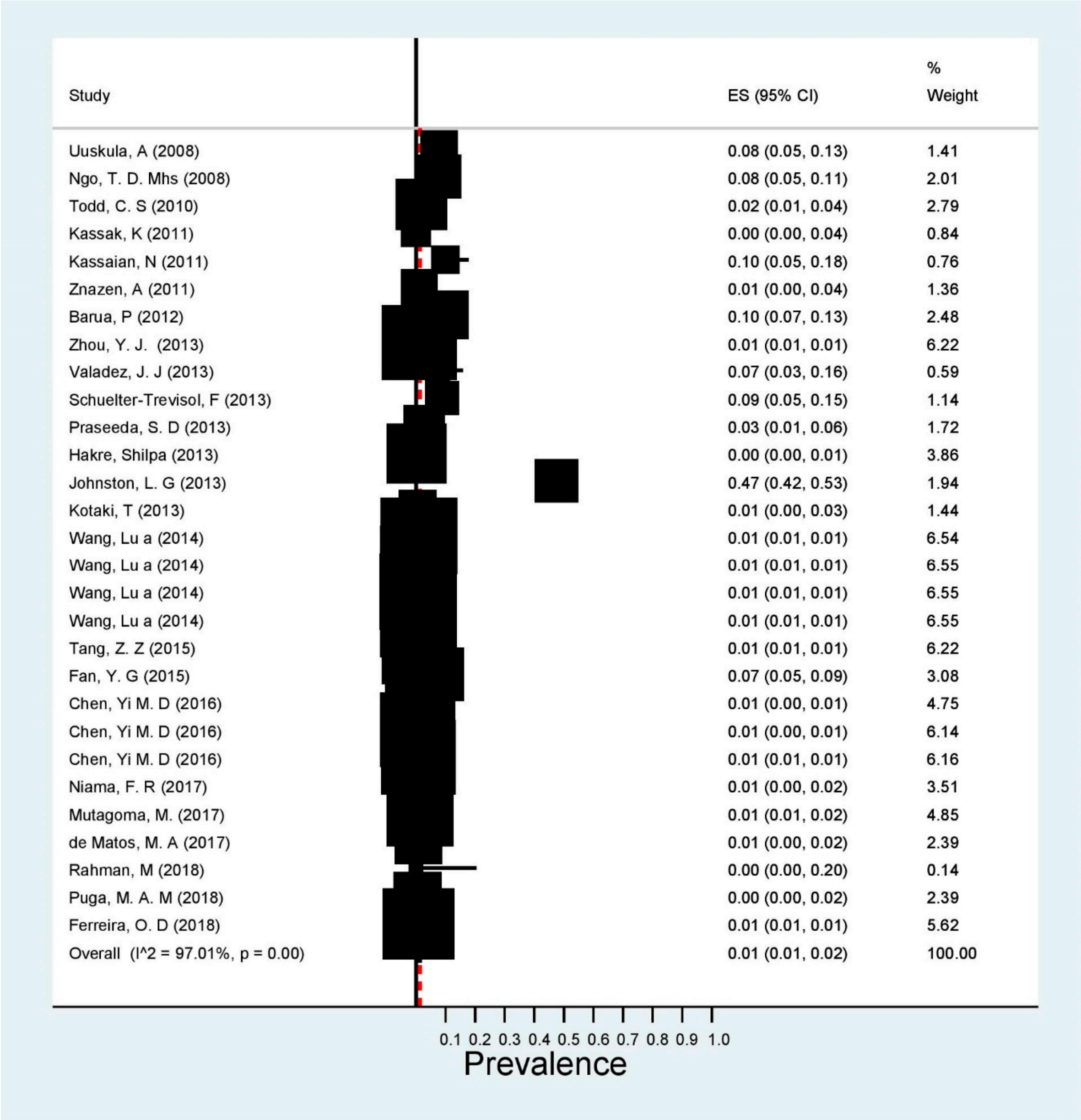

Figure S4. Global prevalence of HCV among FSWs worldwide; 2008–2018.

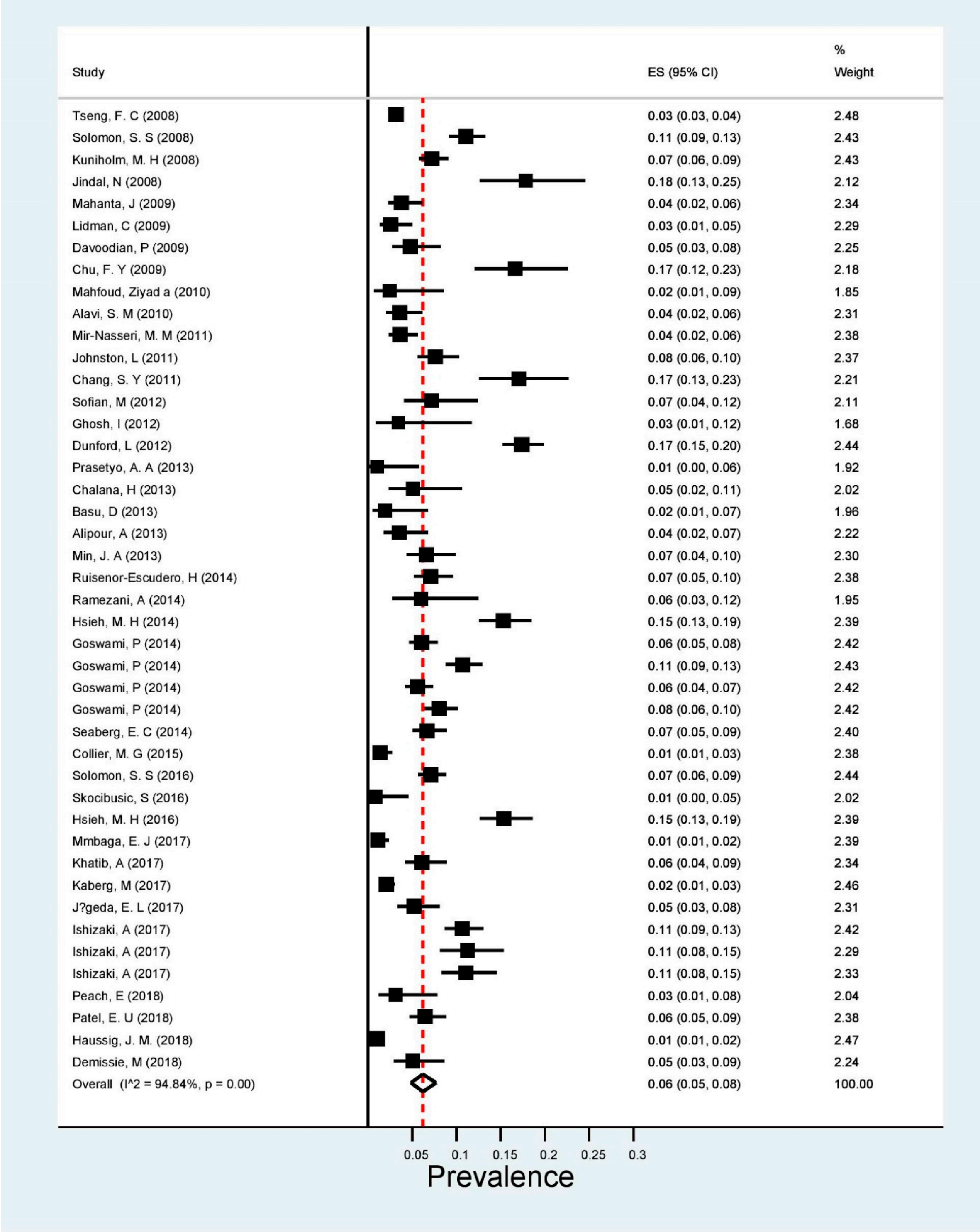

Figure S5. Global prevalence of HBV among PWID worldwide; 2008–2018.

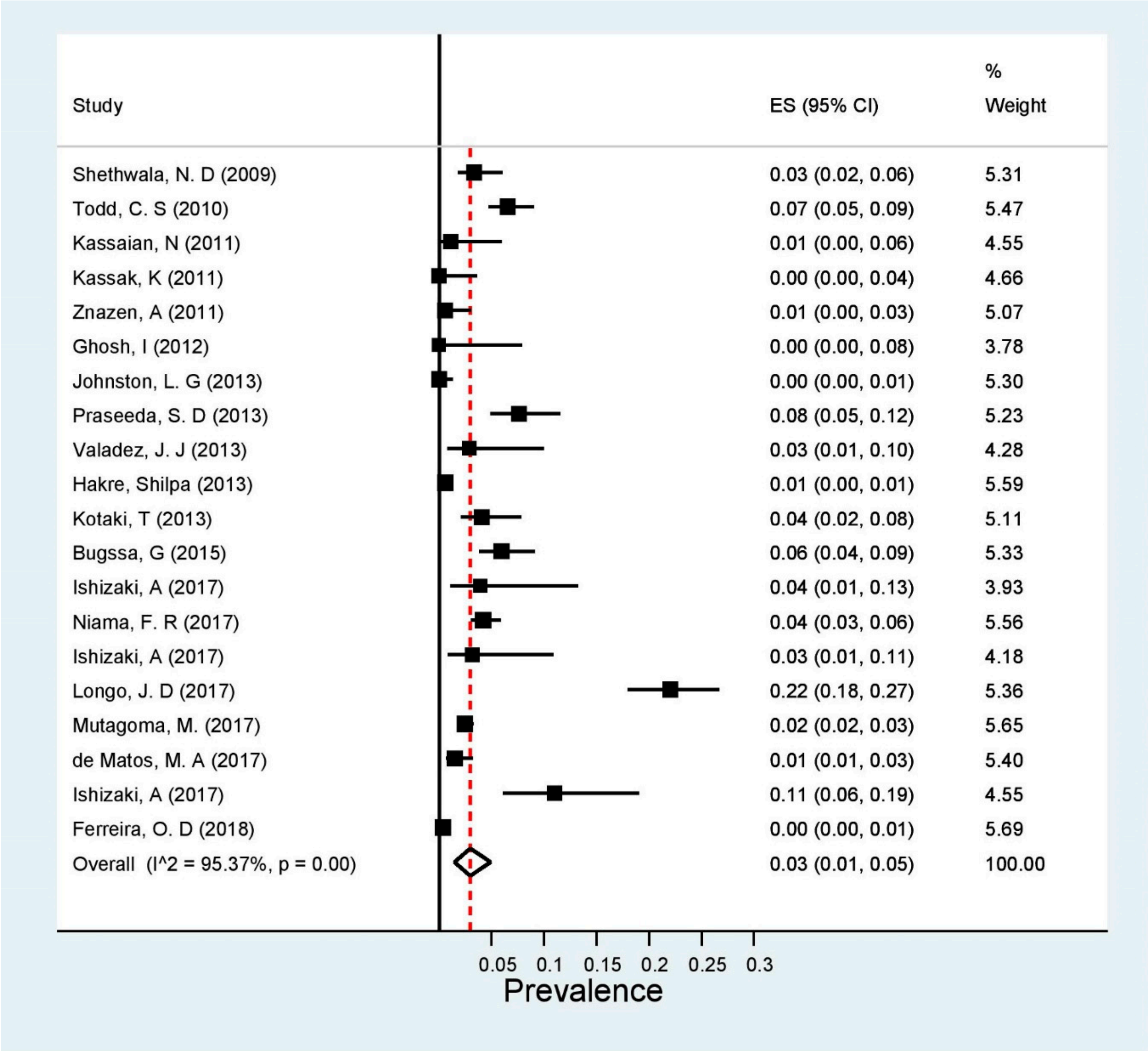

Figure S6. Global prevalence of HBV among FSWs worldwide; 2008–2018.

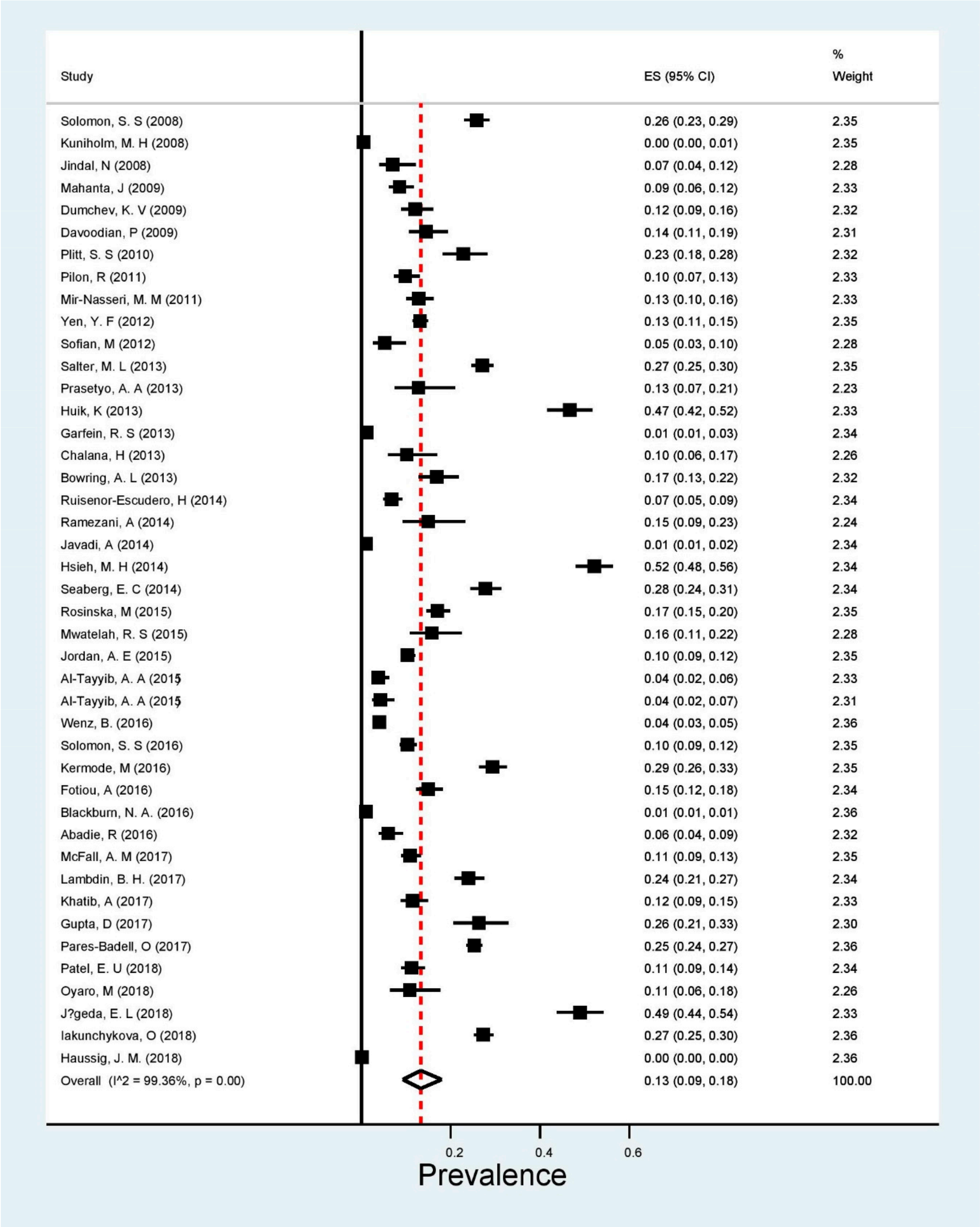

Figure S7. Global prevalence of HIV/HCV co-infection among PWID worldwide; 2008–2018.

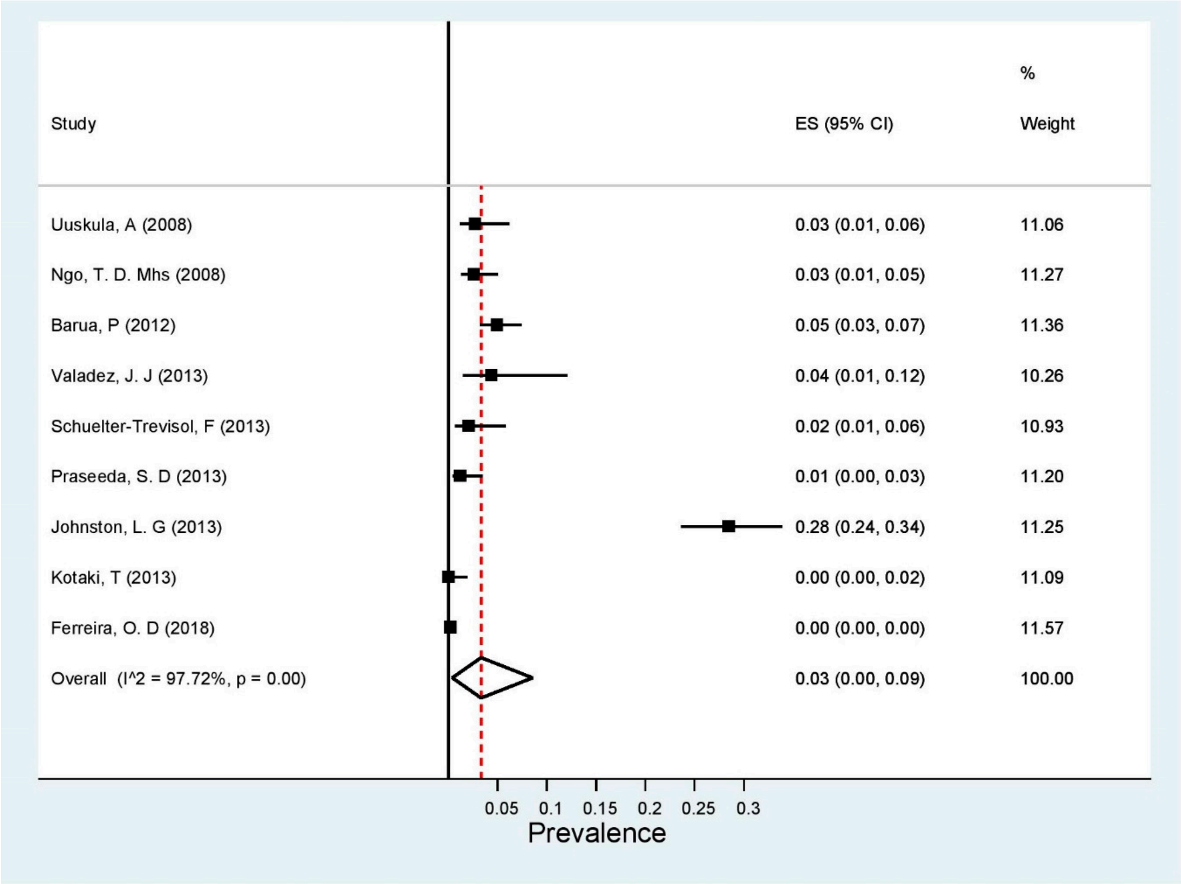

Figure S8. Global prevalence of HIV/HCV co-infection among FSWs worldwide; 2008–2018.

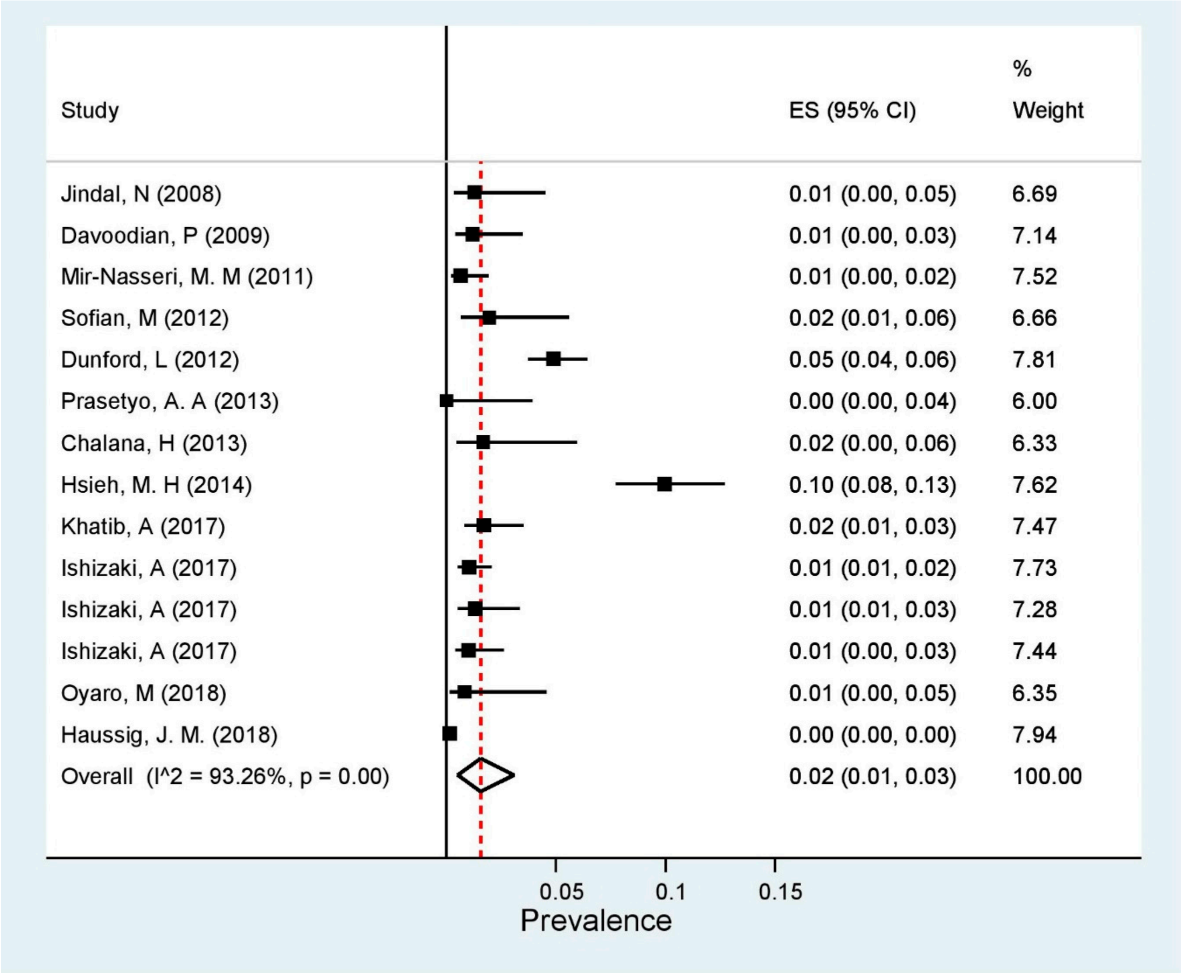

Figure S9. Global prevalence of HIV/HBV co-infection among PWID worldwide; 2008–2018.

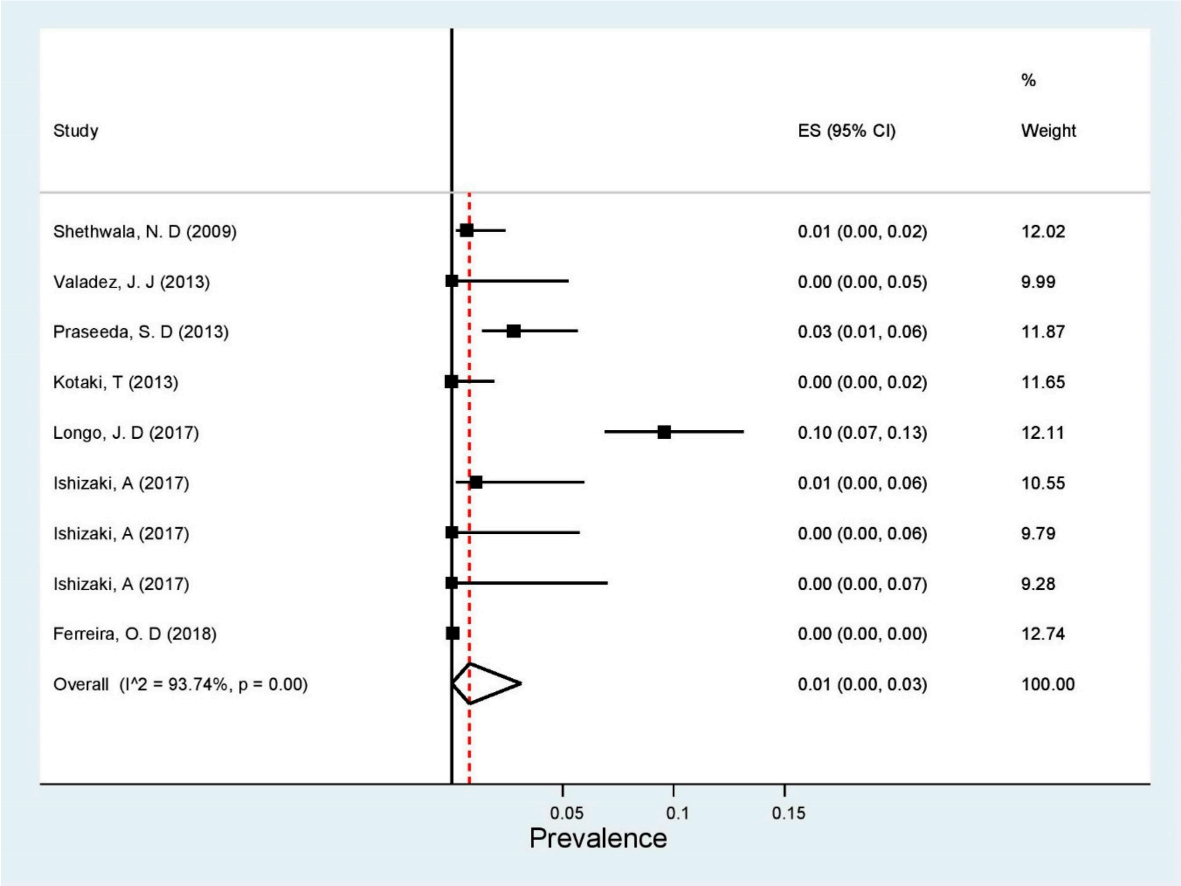

Figure S10. Global prevalence of HIV/HBV co-infection among FSWs worldwide; 2008–2018.

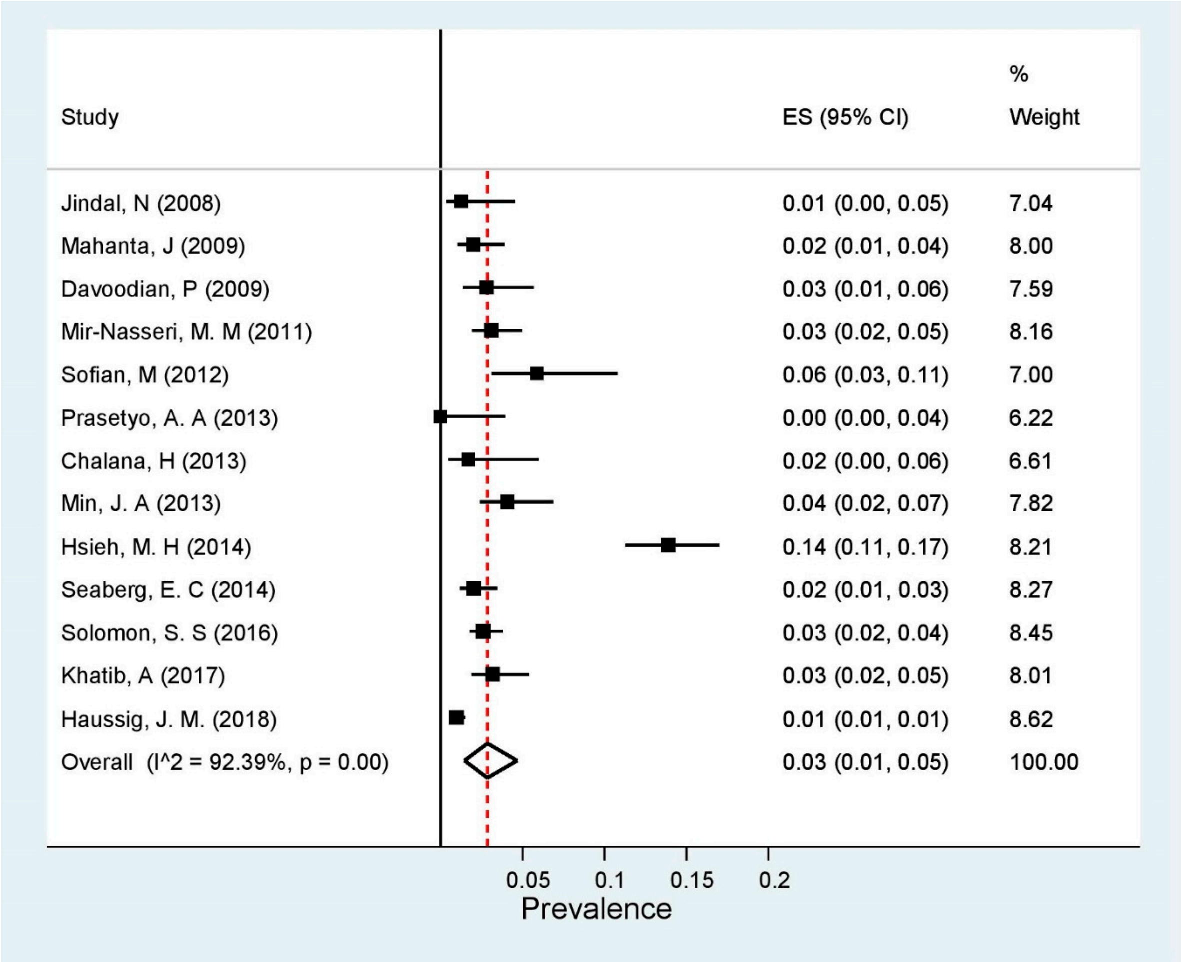

Figure S11. Global prevalence of HCV/HBV co-infection among PWID worldwide; 2008–2018.

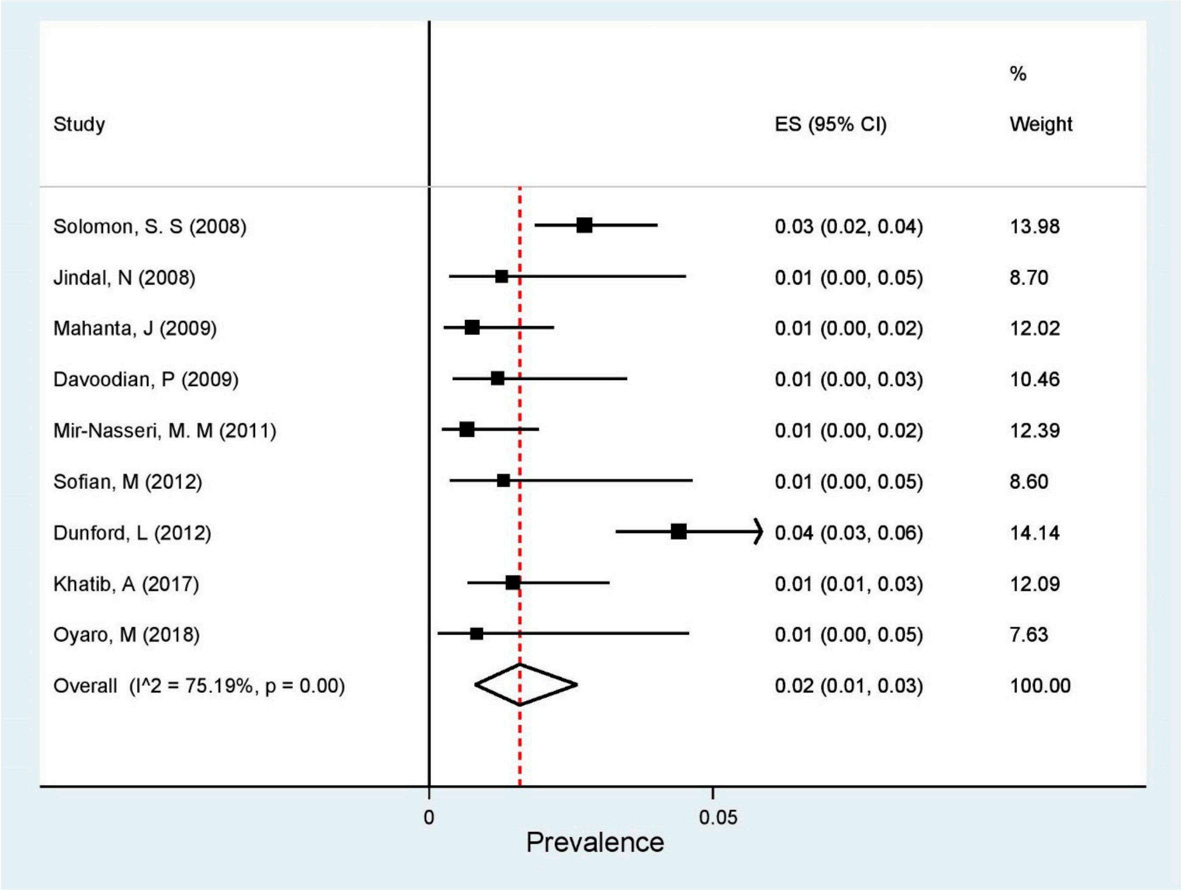

Figure S12. Global prevalence of HIV/HCV/HBV co-infection among PWID worldwide; 2008–2018.
